# Global Mapping of Traditional Chinese Medicine into Bioactivity Space and Pathways Annotation Improves Mechanistic Understanding and Discovers Relationships between Therapeutic Action (Sub)classes

**DOI:** 10.1155/2016/2106465

**Published:** 2016-02-18

**Authors:** Siti Zuraidah Mohamad Zobir, Fazlin Mohd Fauzi, Sonia Liggi, Georgios Drakakis, Xianjun Fu, Tai-Ping Fan, Andreas Bender

**Affiliations:** ^1^Centre for Molecular Science Informatics, Department of Chemistry, University of Cambridge, Lensfield Road, Cambridge CB2 1EW, UK; ^2^Malaysian Institute of Pharmaceuticals and Nutraceuticals (IPharm), Ministry of Science, Technology and Innovation, 11800 Penang, Malaysia; ^3^Department of Pharmacology and Chemistry, Faculty of Pharmacy, Universiti Teknologi MARA, Puncak Alam Campus, 42300 Bandar Puncak Alam, Selangor, Malaysia; ^4^Department of Biomedical Sciences, University of Cagliari, Cittadella Universitaria di Monserrato, SP 8, 09042 Monserrato, Italy; ^5^School of Chemical Engineering, National Technical University of Athens, Zografou Campus, 9 Heroon Polytechniou Street, 15780 Athens, Greece; ^6^School of Information Management, Shandong University of Traditional Chinese Medicine, Jinan 250355, China; ^7^Department of Pharmacology, University of Cambridge, Tennis Court Road, Cambridge CB2 1PD, UK

## Abstract

Traditional Chinese medicine (TCM) still needs more scientific rationale to be proven for it to be accepted further in the West. We are now in the position to propose computational hypotheses for the mode-of-actions (MOAs) of 45 TCM therapeutic action (sub)classes from* in silico* target prediction algorithms, whose target was later annotated with Kyoto Encyclopedia of Genes and Genomes pathway, and to discover the relationship between them by generating a hierarchical clustering. The results of 10,749 TCM compounds showed 183 enriched targets and 99 enriched pathways from Estimation Score ≤ 0 and ≥ 5% of compounds/targets in a (sub)class. The MOA of a (sub)class was established from supporting literature. Overall, the most frequent top three enriched targets/pathways were immune-related targets such as tyrosine-protein phosphatase nonreceptor type 2 (PTPN2) and digestive system such as mineral absorption. We found two major protein families, G-protein coupled receptor (GPCR), and protein kinase family contributed to the diversity of the bioactivity space, while digestive system was consistently annotated pathway motif, which agreed with the important treatment principle of TCM, “the foundation of acquired constitution” that includes spleen and stomach. In short, the TCM (sub)classes, in many cases share similar targets/pathways despite having different indications.

## 1. Introduction

Traditional Chinese medicine (TCM) has been practiced for thousands of years for the prevention and treatment of diseases using a unique system of theory, diagnosis, and treatment [1, 2]. The philosophical background of TCM is based on Yin and Yang, as well as the Five Elements (agents) theories. The Yin and Yang are the harmony of two opposite energies and the Five Elements describe the five interdependent functional organs, heart, liver, spleen, lung, and kidney, with each organ's own Yin and Yang [3, 4]. When a human body suffers from a disease, the dynamic balance and the relationship of the Five Elements are disturbed; hence, to rectify the disturbance, TCM applies a holistic approach with the key therapeutic principles being “*Zheng*” (meaning syndrome or pathological patterns seen in patients) and “*Fufang*” or “*Fang Ji*” (meaning compound formulations consisting of materia medica) [5–11]. The Chinese medicines can be organized into several classifications such as therapeutic actions, source of the medicine, and internal organs [12]. In this study, the classification of the Chinese medicines follows the therapeutic actions, of which some also possess subclassifications based on clinical applications recorded by TCM monographs (Table 1) [13]. A combination of two or more Chinese medicine categories makes up a treatment formula, which then contains a considerable number of chemical compounds [14].

The mixture of compounds in the formula works through the therapeutic principle* Jun-Chen-Zuo-Shi*, by maximizing the therapeutic effects and minimizing the side effects [1, 15, 16]. Based on the healing/pharmacological properties and constituents of each medicine, the Jun (emperor) component is the principal phytocomplex targeting the major symptom of the disease. There are only a few varieties of Jun medicinal that are administered as a single formula, usually in large doses. The Chen (minister) components synergize with Jun to strengthen its therapeutic effects and may also treat secondary symptoms. The Zuo (assistant) medicinal reduces or eliminates possible adverse or toxic effects of the Jun and/or Chen components, while also enhancing their effects and sometimes treating secondary symptoms. Finally, the Shi (courier) components facilitate delivery of the principal components to the lesion sites or facilitate the overall action of the other components [17, 18]. Therefore, at the molecular level, a TCM formula, which is a multicomponent and multitarget agent, is assumed to modulate a series of protein targets in an integrative manner to harmonise the body system [19]. In brief, TCM is a well-structured system from diagnosis to healing, whose theories and medicines are rationally connected and interdependent. Note that a TCM* Fufang* is primarily based on medicinal plants but may also contain fungi (e.g.,* Ganoderma lucidum*), mineral (realgar), and occasionally animal products (e.g.,* Calculus bovis*).

TCM compounds derives from the biodiversity of natural products, of which is a rich resource for discovering new TCM-based drugs. However, to develop a novel TCM-based drug still remains challenging. One of the factors that contributes to making it a challenge is an undefined medicine concoction, where characterising the complex formulation by using methods to isolate the compounds is an exhaustive task that is very time-consuming [20, 21]. Although many compounds have been isolated from Chinese herbals [22, 23], their modes-of-action (MOAs), in many cases, are still not yet understood at the molecular level [21]. Another challenging issue in TCM is measuring the efficacy, pharmacokinetic-pharmacodynamic profiles, and dose-efficacy relationship of multiple compounds simultaneously, both* in vitro* and* in vivo* [20, 21, 24]. However, as shown in this work, particularly related to MOAs, it is now possible to suggest the MOA of TCM compounds using* in silico* target prediction, hence providing testable hypotheses to guide towards finding new molecular entities.

Various methods are available for* in silico* target prediction such as chemical similarity searching [25], analysis of “bioactivity spectra” [26, 27], data mining in annotated chemical database [28, 29], and protein panel docking [30, 31]. Protein panel docking is one of the earliest tools that has been widely used in TCM, where a compound is docked to a wide panel of potential proteins and the proteins are subsequently ranked based on calculated binding affinity scores [32]. Ehrman et al. employed pharmacophore-assisted docking to screen TCM compounds active against inflammation against four protein targets, which were cyclooxygenases (COX) 1 and 2, p38 MAP kinase (p38), c-Jun terminal-NH2 kinase, and type 4 cAMP-specific phosphodiesterase (PDE4) [33]. In a different study, where the* in silico* target prediction was further validated experimentally, Zhang et al. identified putative targets of 19 natural products from two medicinal plants in TCM which were used for the treatment of diabetes and inflammation using a reverse docking approach, TarFisDock server [34]. The natural products showed moderate inhibitory activities against the most frequent target candidate, dipeptidyl peptidase IV (DPP-IV), with IC_50_ values ranging from 14.14 *μ*M to 113.76 *μ*M in an* in vitro* enzyme assay [34]. Although protein panel docking method requires only the chemical structures of the putative active ingredients, it is limited to high quality protein structures and the accuracy of the docking programs used [35].

**Figure 1:**
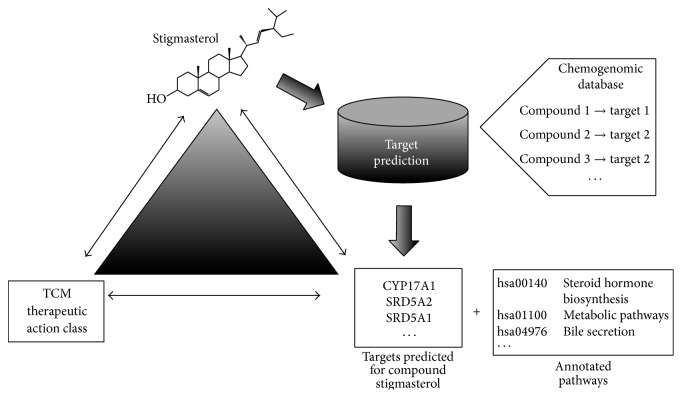
Visualization of the link between orphan compounds taken from TCM databases, predicted targets, and TCM medicinal subclasses. The mode-of-action of compounds in TCM can be hypothesised* via* an* in silico* target prediction algorithm. A predicted target can furthermore be annotated with pathways, which could provide a better insight into the compound's MOA.

In this study, the methodology opted for was the ligand-based target prediction based on large bioactivity database available. Data mining in annotated chemical databases was used to predict the protein targets of TCM compounds to suggest their MOAs. This method becomes viable due to the increasing availability of bioactivity databases [36, 37]. The target prediction algorithm consists of small molecule databases annotated with bioactivity data to map “chemical space” onto “biological activity space.” Based on the principle of molecular similarity, the method, by generating a statistical model using the available data, measures the likelihood of an orphan compound to modulate a target [38]. The model infers the ligand-target modulation based on the molecular similarity, which can suggest the MOA of an orphan compound of TCM by associating the target with the known phenotypic effects of the compound (Figure 1).

Related methods have been developed before and applied in various different settings. One of the earliest studies published on target prediction is the Prediction of Bioactivity Spectra for Substances (PASS) method [39]. This method, which was developed using structure-activity relationships for more than 300,000 biological active compounds in its training set, allows the prediction of more than 4,000 biological activities, based on the structural formula of a compound, with an average accuracy above 95% in the internal model evaluation. Nidhi et al. used a large chemogenomic dataset to develop the* in silico* target prediction [28]. In this work, compound-target pairs from the WOrld of Molecular BioAcTivity (WOMBAT) database were extracted and models trained on extended-connectivity fingerprints to predict the most likely targets for all compounds from a different database, the MDL Drug Database Report (MDDR). The results showed that, on average for 10 test cases, the top three targets were predicted correctly for 77% of the compounds. In the Similarity Ensemble Approach (SEA), the target prediction was developed by grouping and relating the protein targets based on chemical similarity among their ligands, which were quantitatively measured using an algorithm adapted from BLAST [40]. The study discovered that the chemical structure of methadone showed close structural similarity to ligands for the M3 muscarinic receptor, and the prediction was validated experimentally. In TCM, this method has also been applied to study either TCM compounds in general or, specifically, compounds from TCM formulations.* In silico* target prediction was developed by integrating chemical, genomic, and pharmacological approaches using Random Forest and Support Vector Machine with sensitivity of 81.33% and specificity of 93.62% for internal validation [41]. This model was applied by Li et al. to study the MOA of Chinese herbs for the treatment of cardiovascular disease, with the predicted targets being further validated by molecular docking approach [42]. The model was also applied to predict proteins for TCM formulations, Xiao-Chaihu-Decoction and Da-Chaihu-Decoction, with their MOAs being verified by building network pharmacology [43].

The motivation of the current study is prompted by the success to link TCM and Ayurveda compounds to the predicted targets that were relevant for the indications of various therapeutic action classes in TCM and cancer in Ayurveda [44]. In the study, an* in silico* target prediction method using the Naïve Bayes Classifier was able to associate TCM compounds from the “tonifying and replenishing” therapeutic action class, for example, with their known phenotype, hypoglycaemic effect, through the predicted targets such as sodium glucose cotransporter 1 (SGLT1), SGLT2, and protein tyrosine phosphatase to explain their MOA. In addition, in few cases, the predicted targets led to novel MOA and side effects being G-protein bile acid receptor 1 (GPBAR1), predicted from Ayurveda compounds, which contributes to cancer pathogenesis through the apoptosis and cell proliferation signalling pathway. The aim of the study was to extend the analysis of the targets prediction compounds of TCM therapeutic action classes including their subclasses by first generating hierarchical clustering. The first part of the study was to understand the MOAs of the subclasses from the predicted targets as well as from annotated KEGG pathways because target alone is insufficient to provide a full biological profile towards the effect of the ligand on a biological system [45, 46]. In the second part, bioactivity spaces of all therapeutic action (sub)classes were compared in order to understand relationships between the clusters. Hence, the global mapping of TCM compounds explored in this work, based on the therapeutic action (sub)classes, not only does provide better insight of the MOAs of the TCM compounds but also describes for the first time the relationship between the therapeutic action (sub)classes.

## 2. Materials and Methods

### 2.1. Dataset and Dataset Preparation

TCM compounds were obtained from TCM Database@Taiwan [13] in SD format. A total of 13,091 compounds from 46 different therapeutic action (sub)classes were imported into MOE [47]. To prepare structures for further analysis, covalently bound Group I metals were disconnected into ionic representation, while keeping only the largest molecular fragments, neutralising the compound, and adjusting the hydrogen and partial charges using MMFF94 (modified) partial charges. The duplicates from each therapeutic action (sub)class were removed, resulting in a total number of 10,749 distinct compounds. The list of classes with the final number of compounds for each (sub)class is provided in Table 1.

### 2.2. Target Prediction

The processed molecular data was then subjected to target prediction that was modelled using the Laplacian-modified Naïve Bayes Classifier; the details of the target prediction algorithm can be found in [48]. Briefly, this model contained 189,147 ligand-protein pairs extracted from CHEMBL [49] v.14.0 across 477 human targets which was used as the training set. The training set contained active compounds with reported activities (*K*_*i*_/*K*_*d*_/IC_50_/EC_50_) of at least 10 *μ*M with a confidence score of 8 or 9 and at least 20 compounds were available to associate the chemical features with a target class.

The molecular descriptors of the compounds were represented by Molprint2D circular fingerprints which have been shown to relate chemical structural space and bioactivity space well with each other [50]. The targets predicted for the new compound use the Naïve Bayes Classifier as a method for classification as follows [51]:

(1) $$\begin{array}{c} P\left(\;\omega_{\alpha }\;|\;\varkappa \;\right)=\frac{P\left(\varkappa \;|\;\omega_{\alpha }\right)P\left(\omega_{\alpha }\right)}{P\left(\varkappa \right)}. \end{array}$$

Here, the probability of a new compound belonging to a target class, *ω*_*α*_, with a given vector molecular feature, *ϰ*, is calculated. The prior target class probability, *P*(*ω*_*α*_), is assumed to be equal in proportion to the molecules, *Nω*, which modulate that class from the total number of molecules, *N*, in the training set. Hence,

(2) $$\begin{array}{c} P\left(\;C=\omega \;\right)=\frac{N\omega_{\alpha }}{N}. \end{array}$$

The denominator, *P*(*ϰ*), calculates the sum of the fractions of molecules from each class from *β* = 1 to *L* in the training set multiplied by the probability of the vector of molecular feature given the target class:

(3) $$\begin{array}{c} \overset{L}{\underset{\beta =1}{\sum }}\frac{N\omega_{\beta }}{N}P\left(\;\varkappa \;|\;\omega_{\beta }\;\right). \end{array}$$

The posterior probability, *P*(*ϰ*∣*ω*_*α*_), is the likelihood of the feature, *ϰ*, given the class, *ω*_*α*_.

The internal validation of the* in silico* target prediction was measured using the 5-fold cross validation with a recall of correct targets larger than 80% in the top 1% of predictions. In the external validation, the algorithm showed a recall of 63.6% in the top 1% of predictions using dataset extracted from WOrld of Molecular BioAcTivity version 2011.1 (WOMBAT) [52].

Only target scores above a confidence score, which are defined individually for each target class, were taken as the output. The confidence score for each class was calculated by the optimal balanced accuracy (precision and recall tradeoff) on a per-target class basis and was used to retain protein targets likely to interact with the compounds in the dataset [53].

### 2.3. Pathway Annotation

Each predicted target was annotated with its full set of pathways from KEGG biological pathways (release 58.1) [54]. It was possible to annotate 405 out of 477 targets with KEGG biological pathways [55, 56].

### 2.4. Enrichment Calculations

To normalise the classification results from target prediction/pathway annotation, enrichment calculations were performed by normalising frequencies of the target prediction/pathway annotation of each therapeutic action subclass of compound to a background of 10,000 compounds that were selected randomly from PubChem [57] and ZINC [58], which consists of 194,849 compounds in total. Two methods were used to perform enrichment calculation in this step, namely, Estimation Score and Average Ratio. The calculation of the scores was performed as follows [55, 56].

#### 2.4.1. Estimation Score

The Estimation Score is based on the frequency on the number of predicted targets/pathways in the random dataset larger than or equal to the frequency on the number of predicted targets/pathways in the test dataset. The absolute frequency (*C* in (4)) was divided by the total number of random datasets, giving a value between 0 (enriched) and 1 (random). Hence,

(4) $$\begin{array}{c} \text{Estimation Score}=\frac{C}{1000}. \end{array}$$

#### 2.4.2. Average Ratio

The Average Ratio is calculated by the ratio of the frequency (*F*) of predicted target/annotated pathway in each random dataset with the frequency (*F*) of predicted targets in the test dataset:

(5) $$\begin{array}{c} \text{Average Ratio}=\frac{F\left(\text{random set1}\right)/F\left(\text{test set}\right)+F\left(\text{random set2}\right)/F\left(\text{test set}\right)+\cdots +F\left(\text{random set10000}\right)/F\left(\text{test set}\right)}{10000}. \end{array}$$

In this study, enriched targets/pathways were considered if they showed an Estimation Score ≤ 0.01, and descending Average Ratio was used to further discriminate important targets in agreement with previous work by Liggi et al. [55, 56]. The relative cutoff of both target frequency (TF)/pathway frequency (PF) that was ≥5% of the highest predicted target/pathway frequency was used after ranking the targets using the Estimation Score and Average Ratio methods to determine which targets were considered to be enriched in particular therapeutic action (sub)classes.

### 2.5. Hierarchical Clustering Based on the Bioactivity Space of the Therapeutic Action (Sub)classes

The frequencies of compounds across 477 targets for each therapeutic action (sub)class were subjected to agglomerative hierarchical clustering [59, 60]. The clustering method involved two steps as follows.

#### 2.5.1. Selecting Measures of Dissimilarity or Similarity

The dissimilarity distance between two therapeutic action (sub)classes was calculated using the “dist” function in *R* [61] using the “Euclidean” method after scaling the frequencies.

#### 2.5.2. Clustering

Clustering was performed using the “hclust” function in *R* [61] based on the previously calculated Euclidean distance and Ward's clustering method [62]. In this method, two clusters were merged if the sum square Euclidean distance was minimal.

A cutoff dissimilarity distance of approximately 20 was applied in order to obtain a manageable number of clusters, defining 14 groups of therapeutic action (sub)classes, namely, clusters I to XIV.

### 2.6. Targets and Pathways Analysis

The top three enriched targets/pathways were inspected with regard to their ability to explain the MOA of the compounds classified in the therapeutic action (sub)classes. To improve the mechanistic understanding of the MOA, the top three enriched targets/pathways were linked to the phenotypes of the (sub)classes with supporting evidence from the literature and supporting* in vitro* or* in vivo* studies of the Chinese medicines' extracts or isolated compounds. However, supporting* in vitro* and* in vivo* studies were excluded in the pathway analysis because, in many (sub)classes, no information was found. The 14 clusters were grouped based on the number of (sub)classes in a cluster, which ranged from ten (sub)classes to only one (sub)class that derived from different classes. Three clusters were analysed in detail in the next section. Cluster VII was the only cluster composed of (sub)classes with the same TCM vital substance of its meridian system, blood. Cluster X was picked as a representative of a cluster of different classes while cluster XII was selected as a representative of a cluster with only one type of (sub)class. The top three enriched targets/pathways per selected (sub)classes were summarized in Tables 3 and 4 and the top three enriched targets/pathways of all (sub)classes can be found in the Supplementary Material available online at http://dx.doi.org/10.1155/2016/2106465.

To compare the bioactivity spaces among the clusters, all enriched targets in a cluster were classified according to their protein family as derived from UniProt [63]. The enriched pathways for all clusters were also classified according to KEGG ortholog groups, which are derived by comparing sequence similarity of individual genes and defining the functional group from the list of genes in the respective group [45]. The KEGG ortholog group will be called a pathway motif onwards. All the enriched targets were annotated from 59 protein families and the enriched pathways were annotated from 33 pathways motifs. A major protein family/pathway motif for a cluster was defined if the number of enriched targets/pathways was at least 5% of the total number of enriched targets/pathways in the respective cluster and it was present in at least eight clusters. Only five major protein families and eight major pathway motifs were identified. The frequencies of enriched targets/pathways per cluster were normalised before constructing two different heatmaps using heatmap.2 function of gplots package in *R* [61], in order to visualize whether the major protein families/pathway motifs were equally important across clusters.

## 3. Results

### 3.1. Target Prediction and Pathway Annotation

The* in silico* target prediction of 10,749 TCM compounds yielded 409 unique targets, of which 183 were enriched targets. In the pathway annotation, the total number of unique pathways was 171, of which 99 were enriched pathways. The results discussed from here onwards cover 45 of the 46 therapeutic action (sub)classes only. One therapeutic action subclass is not included because no target was retained from the “Tranquilizing-Settling” (Tranquil-settle) subclass, which contained only one compound. This subclass was therefore omitted from hierarchical clustering and all subsequent analyses.

### 3.2. Hierarchical Clustering

**Figure 2:**
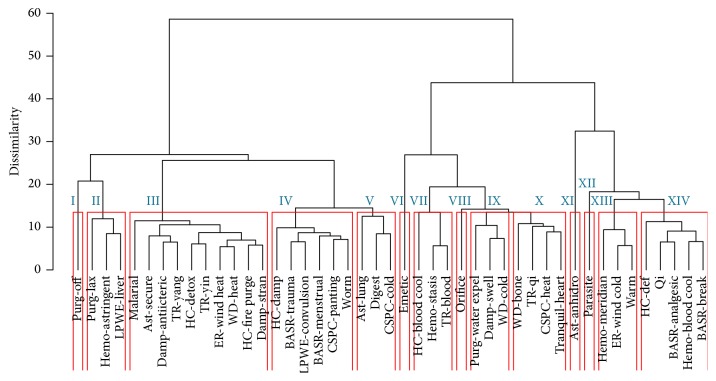
Hierarchical clustering analysis of TCM classes and subclasses is based on the similarity of the bioactivity fingerprint of each class. The “Tranquil-settle” subclass was not included here (and in the further analysis) since it only contained a single compound for which no reliable targets could be predicted. The (sub)classes were defined into 14 clusters, where clusters VII, X, and XII were selected for further analysis based on the top three enriched targets/pathways.

In Figure 2, a dendrogram shows the hierarchical clustering of 45 TCM therapeutic action (sub)classes based on their bioactivity fingerprints. The cluster tree generates diverse spread of the 45 therapeutic action (sub)classes, which is defined into 14 clusters. In many instances, branches of the dendrogram paired up from different types of classes or subclasses. Based on the molecular similarity principle [64], this observation indicates that many similar compounds are present in both (sub)classes despite having different therapeutic actions. The link of the top three enriched targets of the therapeutic action (sub)classes is discussed in the next sections as well as their top three enriched pathways.

### 3.3. Target Analysis

#### 3.3.1. Clusters with Four Therapeutic Action (Sub)classes (Cluster X)

Only cluster X represents this group. The subclasses in cluster X are “wind-dampness dispelling, bone (sinew) strengthening” (WD-bone), “tonifying and replenishing, qi tonifying” (TR-qi), “cough suppressing and panting-calming, clearing and heat phlegm resolving” (CSPC-heat), and “tranquilizing, heat nourishing tranquilizing” (Tranquil-heart). The top three enriched targets from cluster X are mainly implicated in immunomodulation, namely, steryl-sulfatase (STS) [65], tyrosine-protein phosphatase nonreceptor type 2 (PTPN2) [66], and peptidyl-prolyl cis-trans isomerase FKBP1A (FKB1A) [67], glucose homeostasis, such as sodium glucose transporter 1 (SGLT1) and sodium glucose transporter 2 (SGLT2) [68], cancer, such as DNA topoisomerase 1 (TOPO1) [69], reproductive system, such as testosterone 17-beta-dehydrogenase 3 (17-beta-HSD 3) [70], and central nervous system (CNS), such as glutamate carboxypeptidase 2 (CGPII) [71].

Dissecting the* in silico* target prediction per subclass, starting from the “wind-dampness dispelling, bone strengthening” (WD-bone), the top three enriched targets are TOPO1 (Estimation Score (ES) = 0, Average Ratio (AR) = 0.0144), SGLT1 (ES = 0, AR = 0.0342), and STS (ES = 0, AR = 0.0370). From the* in silico* target prediction, acankoreosides A–C derived from* Acanthopanax gracilistylus* were suggested to modulate TOPO1 and SGLT1 in the subclass, while compounds from* Homalomena occulta* such as asperpenoid, bullatantriol, and homalomenol were suggested to modulate STS, as well as compound such as quercetin of* Taxillus chinensis*. The target prediction of compounds from* Acanthopanax gracilistylus* is supported by an* in vitro* study where the herb's extract inhibits cell proliferation of several types of cancer cells [72]. It is also reported that the extract of* Taxillus chinensis* exhibits significant anti-inflammatory activity* in vitro* [73]. The herbs from this class are used to relieve pain, relax muscle and tendons, open channels and collaterals, and strengthen tendons and bones [12, 74]. The actions of the herbs are related to the disturbance of muscular function in diabetes [75] and cancer cachexia which affects protein and lipid metabolism in skeletal muscle [76].

The top three enriched targets for “tonifying and replenishing, qi tonifying” (TR-qi) subclass are PTPN2 (ES = 0, AR = 0.0174), SGLT2 (ES = 0, AR = 0.0282), and SGLT1 (ES = 0, AR = 0.0321). Many compounds from* Glycyrrhiza glabra*,* Glycyrrhiza uralensis*,* Dolichos lablab*,* Panax ginseng*, and* Astragalus membranaceus* were predicted to modulate PTPN2 and SGLT1. In particular to PTPN2 [77], a compound, glycyrrhizin from* Glycyrrhiza glabra* and* Glycyrrhiza uralensis*, is supported by an* in vivo* study that suggests the compound ameliorates all established chronic histopathologic changes of lung tissue in the mouse model of asthma [78]. TCM describes the notion that the medicines from this subclass act on the spleen and lung, in which the deficiency of lung qi is characterised by shortness of breath like in asthma [12].

A subcluster consists of two therapeutic action subclasses seen to have highly similar bioactivity space. The first subclass is “cough suppressing and panting-calming, clearing and heat phlegm resolving” (CSPC-heat) with the top three enriched targets being PTPN2 (ES = 0, AR = 0.0112), TOPO1 (ES = 0, AR = 0.0236), and 17-beta-HSD 3 (ES = 0, AR = 0.0341). Compounds from* Platycodon grandiflorum* such as platycosides A–M were suggested to be modulated by all the top three enriched targets and compounds from* Bambusa tuldoides*,* Peucedanum decursivum*, and* Trichosanthes kirilowii* were mostly predicted to modulate 17-beta-HSD 3. Although* Platycodon grandiflorum* [79] and* Trichosanthes kirilowii* [80] are reported to exhibit anticancer properties, the reports do not support the link between the top three enriched targets and the indication of therapeutic action subclass to rationalise the MOA of the compounds.

The second subclass, “tranquilizing, heart nourishing tranquilizing” (Tranquil-heart), lists FKB1A (ES = 0, AR = 0.0077), PTPN2 (ES = 0, AR = 0.0167), and CGPII (ES = 0, AR = 0.0209) as its top three enriched targets. Compounds of three different herbs were predicted to modulate FKB1A and PTPN2 and only compounds of* Ganoderma lucidum*, such as ganoderic acid and lucidenic acid derivatives, were predicted to modulate CGPII. The prediction of CGPII from triterpenoids of* Ganoderma lucidum* such as ganoderic acids is supported by Zhang et al., where triterpenoids exhibit nerve growth factor or brain-derived neurotrophic factor activities* in vitro*, which has the therapeutic potential in neurodegenerative diseases [81]. The therapeutic actions of the herbs from the subclass are described to have effects on central nervous system [12]. In conclusion, the MOAs of the compounds for three subclasses in cluster X can be suggested from their top three enriched targets.

#### 3.3.2. Clusters with Three Therapeutic Action (Sub)classes (Cluster VII)

Cluster VII is one of the clusters that consists of three therapeutic action subclasses, in which two subclasses, “hemostatic, stasis resolving” (Hemo-stasis) and “tonifying and replenishing, blood” (TR-blood), show highly similar bioactivity space. The third subclass is “heat-clearing, blood cooling” (HC-blood cool). Overall the top three enriched targets in the cluster can be classified into immunomodulation, which are PTPN2 [66], protein kinase C beta type (PKC-*β*) [82], protein kinase C eta type (PKC-*η*) [83], and protein kinase C gamma type (PKC-*γ*) [84], cancer, namely, TOPO1 [69], and glucose homeostasis, such as SGLT2 [68]. The top three enriched targets in “hemostatic, stasis resolving” (Hemo-stasis) and “tonifying and replenishing, blood” (TR-blood) are PTPN2 (ES = 0, AR = 0.0089), PKC-*η* (ES = 0, AR = 0.0182), PKC-*γ* (ES = 0, AR = 0.0209), and PTPN2 (ES = 0, AR = 0.0140), PKC-*β* (ES = 0, AR = 0.0230), and PKC-*ε* (ES = 0, AR = 0.0240), in which all of them are implicated in immunomodulation. In the “hemostatic, stasis resolving” (Hemo-stasis) subclass, compounds such as ginsenosides and notoginsenosides of* Panax notoginseng* were predicted to modulate all top three enriched targets. The target prediction also showed that anthraquinone compounds from* Rubia cordifolia* such as purpurin, ruberythric acid, and soranjidiol modulate PKC-*β* and PKC-*ε*. In support of the target prediction of anthraquinone compounds of the second herb, a study reported that the herb's ethanol extract shows wound healing activities in mice, which from histological evaluations indicate marked infiltration of the inflammatory cells, increased blood vessel formation, and enhanced cells proliferation [85]. This finding agrees with the description of the subclass to stop bleeding [12].

In the “tonifying and replenishing, blood” (TR-blood) subclass, compounds from* Paeonia lactiflora* such as albiflorin, gallotannin, and casuarictin were predicted to modulate PKC-*β* and PKC-*η*. PTPN2 and PKC-*η* were both frequently predicted to be modulated by compounds from* Panax notoginseng* such as notoginsenoside and ginsenoside. It is found that ginsenoside Rg1 of* Panax notoginseng* ameliorates liver damage and suppresses proinflammatory cytokines secretion in concanavalin A-induced hepatitis in mice [86]. This subclass is described to have pharmacological effects on the liver, heart, and spleen and prevent failures of the organs [12]. The top three enriched targets for “heat-clearing, blood cooling” (HC-blood cool) subclass are PKC-*β* (ES = 0, AR = 0.0100), TOPO1 (ES = 0, AR = 0.0123), and SGLT2 (ES = 0, AR = 0.0137). In this subclass, compounds from* Paeonia lactiflora* such as albiflorin, isopaeoniflorin, and benzoylpaeoniflorin were predicted to modulate both PKC-*β* and TOPO1, while SGLT2 was predicted to be modulated by compounds from* Rehmannia glutinosa* such as rehmanniosides A and B. We found that the target prediction of SGLT2 is supported by a study on stachyose extract from* Rehmannia glutinosa*, which shows a significant hypoglycaemic effect in diabetic mice [87]. TCM views the notion that the action of the subclass is to promote the generation of body fluids from excessive heat [12, 74] where the consumption of body fluid is one of the symptoms in diabetes [88] and cancer patients [89]. Altogether, in many instances, the MOAs of the compounds can be explained from the enriched targets and can also be linked to the indications of the (sub)classes.

#### 3.3.3. Cluster with One Therapeutic Action (Sub)class (Cluster XII)

One out of five clusters that has only one therapeutic action class is cluster XII, “parasite destroying, dampness eliminating, and itchiness relieving” (Parasite). The top three enriched targets are dihydrofolate reductase (DHFR), which plays a role in bacterial infection and cancer [90] (ES = 0, AR = 0.0532), DNA-dependent protein kinase catalytic subunit (DNA-PKCs), which is implicated in cancer [91] (ES = 0, AR = 0.0644), and tumour necrosis factor (TNF), which is found to exert activities in cancer, inflammation, and bacterial infection [92, 93] (ES = 0, AR = 0.0687). From the target prediction, compounds from* Allium sativum* were predicted to modulate DHFR, such as allicin, allithiamine, and allyl disulphide. This prediction agrees with a study by Adetumbi et al., from which the extract of* Allium sativum* is found to inhibit the synthesis of proteins, nucleic acids, and lipids in* Candida albicans* where the major component of the herb was allicin [94]. The finding relates to the phenotypes of the subclass, which is to kill and expel parasites and subsequently relieve pain [12, 74]. In brief, the MOAs of the class can be linked to the enriched targets and the indications of the subclass.  Table 2 summarises the top three enriched targets of the therapeutic action (sub)classes.

The above analysis can be summarized into two different views, namely, biological space and chemical space. From the biological space's view, many of the top three enriched targets, regardless of therapeutic action (sub)classes in any of the clusters, are implicated in immunomodulation such as PTPN2, PKCs, FKBP1A, and STS, in which PTPN2 and PKCs were frequently predicted. Both PKC family and PTPN2 are implicated in immunomodulation. The frequency of immune-related targets can be related when TCM balances the immune system regulation by either promoting or suppressing the immune factors [95]. The PKC isoenzymes act as important mediators in immune cellular signalling in T- and B-lymphocytes in acquired immune system [96]. PTPN2 also plays a major role in the transmission of immune cell signalling events [66]. From the chemical space's view, triterpenoid is the most frequent phytochemical (Table 3), which was predicted to modulate few of the top three enriched targets, such as TOPO1, PTPN2, and PKCs. The compounds are found in different herbs such as* Acanthopanax gracilistylus*,* Glycyrrhiza glabra*,* Platycodon grandiflorum*,* Ganoderma lucidum*, and* Panax notoginseng*. To validate the result from the chemogenomic principle that similar targets share similar compounds, a compound, CHEMBL 1986122, from CHEMBL database [49] is most similar to acankoreoside A, with Tanimoto coefficient (TC) value of 0.91 (Table 4). The GI_50_ value of CHEMBL 1986122 was 43.05 nM in tumour cell line growth inhibition assay. Hence, the chemogenomics principle appears to stand (at least for acankoreoside A) because both compounds are implicated in cancer and are structurally similar.

### 3.4. Pathway Analysis

#### 3.4.1. Clusters with Four Therapeutic Action (Sub)classes (Cluster X)

The top three enriched pathways in cluster X are mainly implicated in digestive system (carbohydrate digestion and absorption, bile secretion, and mineral absorption). In this cluster, only one pathway, from the top three enriched pathways, is not classified in digestive system, which is terpenoid backbone biosynthesis.

In the “wind-dampness dispelling, bone strengthening” (WD-bone) subclass, the top three enriched pathways are mineral absorption (ES = 0, AR = 0.0342), carbohydrate digestion and absorption (ES = 0, AR = 0.1427), and bile secretion (ES = 0, AR = 0.2050). To link the mineral absorption pathway to the indication of the subclass, it has been reported that minerals such as calcium are important for bone remodelling to strengthen the bone [97]. In the second pathway, carbohydrate digestion and absorption are associated with the indication of the subclass. Here, a study shows that the low carbohydrate-high fat (LH-FC) diets reduced the bone growth, bone structures, and mechanical properties in mice [98]. However, no study can be found to link bile secretion to the indication of the subclass.

“Tonifying and replenishing, qi tonifying” (TR-qi) subclass has mineral absorption (ES = 0, AR = 0.0321), carbohydrate digestion and absorption (ES = 0, AR = 0.1251), and bile secretion (ES = 0, AR = 0.2678) in the top three enriched pathways. Minerals play vital roles in maintaining the cell functions to optimise health and prevent diseases. The first enriched pathway, mineral absorption, the process of recycling iron in erythrocytes, is materialized in splenic macrophages in the red pulp [99] which agrees with the indication that the subclass which is qi tonifying is related to the maintenance of blood flow within the vessels which is implicated directly from spleen's activity [100]. In the second pathway, no study can be found to link carbohydrate digestion and absorption to the indication of the subclass. Relating to the third enriched pathway, bile secretion, it has been reported that the major components of bile acid, chenodeoxycholic acid and glycochenodeoxycholic acid, can induce cyclooxygenase 2 expression and cell proliferation in esophageal squamous cells, suggesting that bile acids may contribute to the inflammation and mucosal thickening [101]. Another paper demonstrates that bile acids may induce airway fibrosis through the production of TGF-*β*1 and fibroblast proliferation [102]. These findings can be associated with the indication of the subclass, which is related to lung deficiency [12].

The top enriched pathways of “cough suppressing and panting-calming, clearing and heat phlegm resolving” (CSPC-heat) subclass are minerals absorption (ES = 0, AR = 0.0380), carbohydrate digestion and absorption (ES = 0, AR = 0.1572), and bile secretion (ES = 0, AR = 0.1958). Relating to the first enriched pathway, mineral absorption, the abnormal distribution of trace elements such as zinc, selenium, and copper has been reported to aggravate oxidative damage and inflammation in the airways and subsequently decreased the lung's function in asthmatic patient [103], where an asthmatic condition is described as retention of heat phlegm in the lung [104]. In the second enriched pathway, carbohydrate digestion and absorption, the ingestion of carbohydrate is reported to attenuate the migration of T-lymphocytes to the bronchial epithelial cell line when it is infected with the common respiratory pathogen human rhinovirus during strenuous exercise [105]. This effect agrees with the phenotype of the subclass which is to dissolve phlegm upon infection in the lung [12, 74]. However, no strong evidence can be found to link bile secretion to the indication of the subclass.

The “tranquilizing, heart nourishing tranquilizing” (Tranquil-heart) subclass lists mineral absorption (ES = 0, AR = 0.0387), carbohydrate digestion and absorption (ES = 0, AR = 0.1163), and terpenoid backbone biosynthesis (ES = 0, AR = 0.2166). In the first enriched pathway, mineral absorption, it is reported that selenium plays an important role in the brain where its deficiency is implicated in senility and Alzheimer's disease [106]. In the third enriched pathway, terpenoid backbone biosynthesis includes mevalonate and nonmevalonate pathway. The mevalonate pathway is a pathway implicated in cholesterol biosynthesis in the brain and deficiencies in cholesterol metabolism can lead to diseases of the central nervous system (CNS) [107]. Both findings agree with the indication of the subclass, which is the pharmacological effects that are on the central nervous system [12]. Although there is no strong evidence to support the second enriched pathway, carbohydrate digestion and absorption, the two enriched pathways can be linked to the indication of the subclass. In short, in many instances, the top three enriched pathways can be associated with the indications of the (sub)classes to explain their MOAs.

#### 3.4.2. Cluster with Three Therapeutic Action (Sub)classes (Cluster VII)

The top three enriched pathways in the cluster can be classified into digestive system (carbohydrate digestion and absorption, bile secretion, and mineral absorption), cellular communication (tight junction), and membrane transport (ABC transporters). The top three enriched targets in “hemostatic, stasis resolving” (Hemo-stasis) subclass are mineral absorption (ES = 0, AR = 0.0267), carbohydrate digestion and absorption (ES = 0, AR = 0.0987), and bile secretion (ES = 0, AR = 0.1872) and “tonifying and replenishing, blood” (TR-blood) subclass lists mineral absorption (ES = 0, AR = 0.0329), carbohydrate digestion and absorption (ES = 0, AR = 0.1055), and tight junction (ES = 0, AR = 0.2053). In “hemostatic, stasis resolving” (Hemo-stasis) subclass, the mineral absorption pathway can be related to zinc deficiency, which has been reported to delay wound healing [108]. In the bile secretion pathway, bile acids are implicated in platelet inhibition by solubilizing the platelets, in which patients with obstructive jaundice were exposed to abnormal hemostasis due to high level of bile acids [109]. Both enriched pathways are related to the indication of the subclass, which is to achieve hemostasis [12], and no study can be found to associate carbohydrate digestion and absorption pathway with the indication of the subclass. In “tonifying and replenishing, blood” (TR-blood), the first enriched pathway, mineral absorption, can be linked to the indication of the subclass which is to strengthen the heart that controls blood vessel [12], when selenium is reported to be protective against cardiovascular disease by contributing to the production of vasodilatory prostacyclin by the endothelium [106]. No study can be found to support the link between the second pathway, carbohydrate digestion and absorption, and the indication of the subclass. In the third enriched pathway, tight junction, the presence of tight junctions in the bile epitheliums acts as barrier to toxic diffusion of bile into hepatic interstitial tissue, which could impair the organ's function [110]. TCM enriched can be associated with the indication of the subclass, which is to strengthen the function of liver [12].

The top three enriched targets for “heat-clearing, blood cooling” (HC-blood cool) subclass are mineral absorption (ES = 0, AR = 0.0276), carbohydrate digestion and absorption (ES = 0, AR = 0.0753), and ABC transporter (ES = 0, AR = 0.0769). The mineral absorption pathway can be linked to the subclass when a decrease in intracellular magnesium concentration is implicated in type 2 diabetes [111] and the disease, according to TCM, is described as the deficiency of body liquid due to heat syndrome [112]. Similar to previous subclass, no study can be found to link the second enriched pathway, the carbohydrate digestion and absorption, to the indication of the subclass. In the third pathway, ABC transporter, it is reported that the high expression of ABCG5 and ABG8 in hypercholesterolemic condition of the heart is involved in cardiovascular protection by lowering plasma cholesterol level [113]. The pathway can be linked to the indication of the subclass when the herbs are described to act on liver and heart [12]. All in all, in many cases, the top three enriched pathways can be linked to the indications of the subclasses to explain their MOAs.

#### 3.4.3. Cluster with One Therapeutic Action (Sub)class (Cluster XII)

In the “parasite destroying, dampness eliminating, and itchiness relieving” (Parasite) class, the top three enriched pathways are steroid biosynthesis (ES = 0, AR = 0.2530), glycerophospholipid metabolism (ES = 0, AR = 0.2941), and collecting duct acid secretion (ES = 0, AR = 0.3420). The first two pathways, steroid biosynthesis and glycerophospholipid metabolism, are part of lipid metabolism, and many reports have suggested their link to immune response. For instance, the steroid biosynthesis is downregulated by interferon type I upon viral infection [114] and the chlamydia exploits the nutrient-rich host cell cytosol by trafficking the glycerophospholipid from the host cell for survival [115]. In relation to the collecting duct acid secretion pathway, the bacterial infection in the kidney is reported to affect the collecting duct acid secretion because the presence of lipopolysaccharide (LPS) of the bacteria inhibits HCO_3_^−^ absorption [116]. These pathways agree with the functions of the subclass, which is to kill and expel parasites [12, 74]. Altogether, the top enriched pathways can be linked to the indication of the class to explain the MOAs.  Table 5 summarises the top three enriched pathways of the therapeutic action (sub)classes.

The above analysis can be summarized into three observations as follows. Firstly, a type of therapeutic action (sub)class is implicated in different enriched pathways and each pathway is involved in a different disease; secondly, more than one therapeutic action (sub)class is implicated in a pathway that is involved in multiple diseases; and thirdly, more than one therapeutic action (sub)class is implicated in different pathways but involved in only one type of a disease. The first observation is indicated, in many cases, by the (sub)classes in any of the three clusters such as bile secretion and mineral absorption in “tonifying and replenishing” (TR-qi). The top three enriched pathways in a therapeutic action (sub)class are implicated in different pathways and diseases. The modulation of one pathway to a disease could provide a better insight into the (sub)class MOA in the biological system. In the second observation, the mineral absorption in cluster VII and cluster X and carbohydrate digestion and absorption in cluster VII are implicated in various types of diseases. The redundancy of a pathway in the pathogenesis of various diseases implies that a pathway could serve multiple purposes; for instance, the mitogen-activated protein kinase (MAPK) signalling pathway was implicated in inflammation, cancer, cardiovascular dysfunction, and Alzheimer's disease [117]. The third observation shows that different pathways from different therapeutic action subclasses in a cluster are implicated in a type of physiological function, such as the ABC transporter and mineral absorption from “heat-clearing, blood cooling” (HC-blood cool) and “tonifying and replenishing, blood” (TR-blood), respectively, which is implicated in physiological function of the heart. The different pathways for a particular physiological function explain that a pathway does not function alone in the manifestation of a disease but through the interactions of multiple pathways [118], which account for the different clinical symptoms. To put it briefly, the pathways annotation not only is beneficial to suggest the MOAs of the (sub)classes based on their indications, but also shows that one pathway could have manifold functions and multiple pathways contribute to the pathogenesis of a disease. From the TCM perspective, it is suggested that the involvement of multiple pathways in the pathogenesis of a disease explains the complex TCM formulation, which consists of a set of herbs from various therapeutic action (sub)classes.

### 3.5. Comparison of Bioactivity Spaces of Clusters

**Figure 3:**
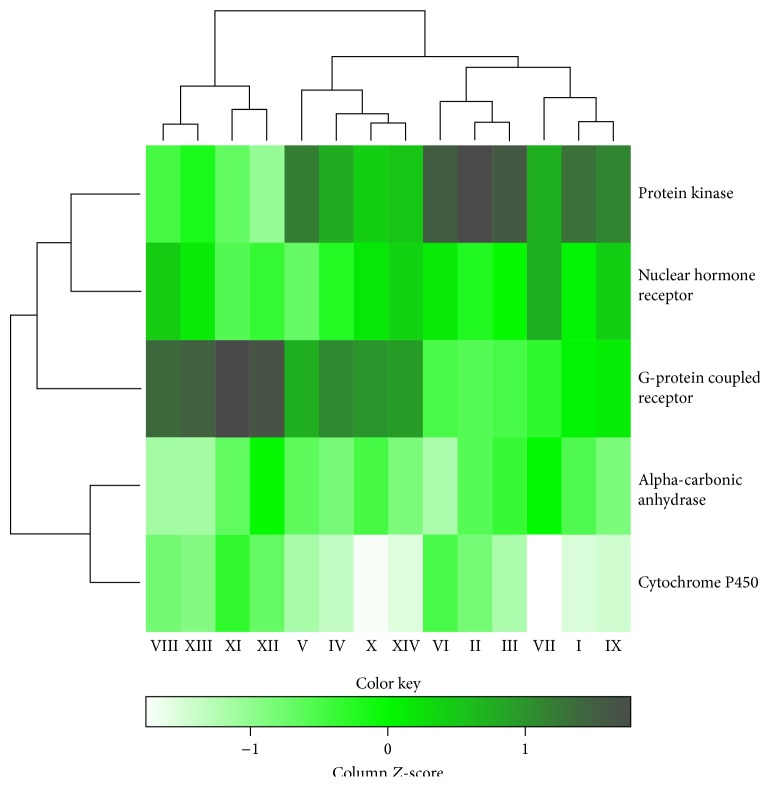
The heatmap compares the five major protein families that were annotated based on the enriched targets in each cluster, which were normalized. The more saturated colour across clusters represents the more significant protein family. GPCR and protein kinase are observed to be significant protein families in almost all clusters. It appears that all the protein families are heavily implicated in the biological processes such as cell regulation, sensory system, and steroid metabolism. The significant cluster for a particular protein family can be suggested to be further explored for a disease with the known protein family such as protein kinase in cancer.

In this part of study, the aim was to investigate the differences of bioactivity spaces among all clusters by classifying all the enriched targets in the cluster to their respective protein families. The 181 enriched targets were classified into 59 protein families. Out of 59 protein families, five protein families were frequently annotated in all clusters, which are G-protein coupled receptor (GPCR), protein kinase, nuclear hormone receptor, carbonic anhydrase, and cytochrome P450. The heatmap in Figure 3 compares the five major protein families that were annotated based on the enriched targets in each cluster. The more saturated colour represents the more significant protein family across all clusters. The numbers of protein families in the clusters were normalised because the distribution of enriched targets was not consistent because numbers of therapeutic action (sub)classes per cluster were different.

What can be seen from the graph is that GPCRs and protein kinases are the most highly classified protein families in almost all clusters. The results were expected as both of the families are the two largest protein families involved in many physiological processes [119, 120], thus explaining why these two protein families were observed to be significant in many of the clusters. In addition, the diversity of the compounds in the (sub)classes also contributed to the prediction of enriched targets from these protein families. At least four protein families were significant across all clusters, except for clusters VII and X in cytochrome P450. The numbers of enriched targets in the cluster were among the lowest across all clusters, and cytochrome P450 was less classified. Nuclear hormone receptors were found to be annotated in all clusters and the frequent predictions of the nuclear hormone receptor family can be explained by the presence of naturally occurring steroids in natural compounds [121]. For the remaining two major protein families, the cytochrome P450 family was also expected to be modulated by most of the subclasses because this protein family plays an important role in the degradation of structurally rather diverse exogenous compounds [122]. The carbonic anhydrase family, which is a ubiquitous enzyme, is involved in the interconversion between carbon dioxide and the bicarbonate ion that is important for many physiological processes [123]. All in all, five major protein families were observed to frequently occur in most of the clusters and were heavily implicated in the biological processes such as cell regulation, sensory system, and steroid metabolism. This analysis has allowed the discovery of the bioactivity spaces connection between subclasses in TCM based on the sets of enriched targets from our* in silico* target prediction in which the compounds from the significant cluster can be further explored for diseases associated with the protein family such as cancer and protein kinase family.

### 3.6. Comparison of Pathways Annotation of Clusters

**Figure 4:**
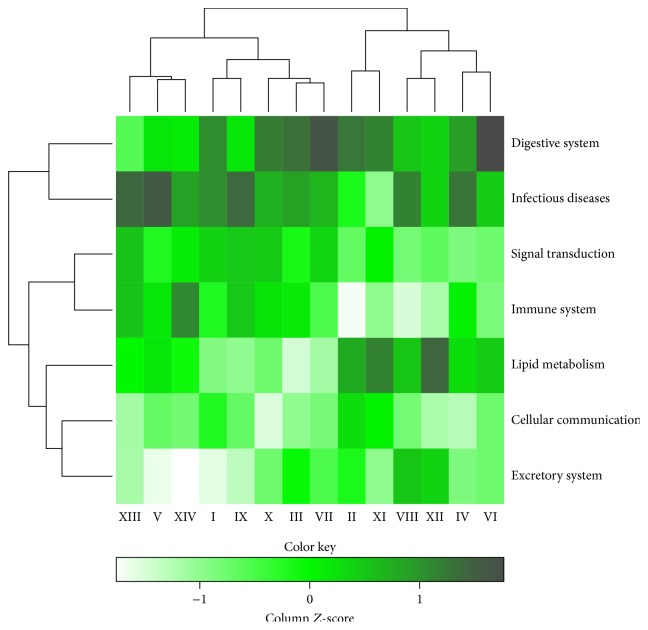
The heatmap compares the seven major pathway motifs that were annotated based on the number of enriched pathways in each cluster, which were normalized. The more saturated colour across clusters represents the more significant pathway motif. The significant cluster for a particular pathway can be suggested to be further explored for a disease with the known pathway motif such as digestive system in liver disease.

In this part of the study, we aimed to investigate the differences of pathway motifs among all clusters by classifying the enriched pathways according to KEGG ortholog. The 99 enriched pathways were classified to 33 pathway motifs, which were almost half the number of total pathway motifs available in KEGG. The major pathway motifs from the classification were infectious diseases, digestive system, immune system, signal transduction, lipid metabolism, cancer, and cellular communication. The heatmap in Figure 4 compares the seven major pathway motifs that were annotated based on the enriched pathways in each cluster. The more saturated colour across clusters represents the more significant pathway motif. The numbers of pathway motifs in the clusters were normalised due to the differences in numbers of the pathways that were enriched among all (sub)classes

In Figure 4, the digestive system is consistently classified in all clusters, where, in many cases, the plots' colours are more saturated compared to other pathway motifs. The digestive system includes the digestion and absorption of macro- and micronutrients and shows that the majority of the enriched pathways are bile secretion, pancreatic secretion, and gastric acid secretion. The bile secretion controls the cholesterol homeostasis by routing the elimination of cholesterol, in addition to harmful exogenous lipophilic substance [124]. In addition to digestive system, infectious diseases, signal transduction, and lipid metabolism are significant pathway motifs in many of the clusters. The significance of infectious diseases can be deduced from frequently predicted immunomodulatory targets as the pathogenic factors are described to attack weakened immune system [125]. The signal transduction pathway motif can be contributed from the classification of one of the major protein families, GPCR, which translates the extracellular signals for the downstream effectors that produce a physiological response in a target cell [126]. Many Chinese medicines have been reported to have lipid regulating effects by influencing the intestinal lipid absorption and lipid metabolism, to name a few [127]. Also, one of the highly observed enriched pathways in the lipid metabolism pathway motif was steroid hormone biosynthesis, which the cytochrome P450 protein family is involved in [128]. It also appears that a few of the pathway motifs are insignificant for some clusters such as immune system for cluster II and excretory system in cluster XIV, which resulted from low number of compounds to influence the targets prediction and pathways annotation. The list of compounds that were annotated for the Chinese medicine in the subclasses might be incomplete to influence the classification of the immune system as well as the remaining missing pathway motifs. In addition, only enriched pathways were used in the classification. Similar to significant clusters for the major protein families, the compounds from significant clusters for the major pathway motifs can be further explored for diseases associated with the protein family such as liver disease and digestive system.

Altogether, this analysis has allowed the discovery of the major pathway motifs in all clusters. Despite having the different therapeutic action (sub)classes in a cluster, in many cases, all the clusters can be classified of having the seven major pathway motifs. The classification of the major pathway motifs was associated with the major protein families analysed in the previous section.

## 4. Discussion

As shown in this study, the global mapping of relationships between TCM therapeutic action classes and subclasses, based on their putative bioactivity spaces and annotated pathways, provides a novel approach to understand the MOAs of TCM formulations. The classification of the enriched targets and pathways according to the protein families and pathway motifs allows the discovery of the relationship between therapeutic action (sub)classes across clusters, defined in the dendrogram. In the first part of the study, we were able to rationalize the link between the top three enriched targets/pathways and the description of the respective therapeutic action (sub)classes. In the bioactivity space, in many cases, the supporting* in vitro* or* in vivo* studies of the herbs' extracts or isolated compounds were also included to illustrate the MOA of the compounds. Among the three most enriched targets, we observed that immunomodulatory targets such as PTPN2 and PKC family were frequently represented across selected therapeutic action classes and subclasses. In TCM, symptoms are usually regarded as the invasion of pathogenic factors, thus sensitizing the immune system to response, and this might provide a mechanistic link between TCM and Western thinking. The other frequently enriched targets were implicated in glucose homeostasis, namely, SGLT1 and SGLT2, as well as cancer, such as TOPO1. The analysis of the enriched pathways showed that the multiple enriched pathways were implicated for one type of disease, which was cardiovascular diseases, and one enriched pathway could be associated with different pathogenesis of diseases such as carbohydrate digestion and absorption and mineral absorption. This finding is in agreement with the utilisation of different herbs in one TCM formulation, in order to modulate biology in the desired polypharmacological manner. In addition, in many cases, the highly annotated pathway motifs are involved in digestive system such as bile secretion, carbohydrate digestion and absorption, and mineral absorption, which can be linked to a theory of “the foundation of acquired constitution” that includes stomach and spleen as the source of production of qi and blood [129].

The dendrogram which was generated based on the* in silico* target prediction of TCM therapeutic action (sub)classes has enabled the visualisation of their bioactivity space, of which, in most of the clusters, the five major protein families were observed to contribute the bioactivity space. The GPCR and the protein kinase family are the two major protein families that contribute to the diversity of the TCM bioactivity space in all clusters. The major KEGG pathways motifs annotated, such as signal transduction and lipid metabolism, were annotated from major protein families, GPCR and cytochrome P450, found in the previous analysis.

Although this study has successfully explained the link of the enriched targets/pathways to their respective therapeutic action (sub)classes and provided a global overview of their bioactivity space, this study is still limited to targets that are only available in the* in silico* target prediction, while the entire human proteome is much larger than 477 targets. Thus, extending the biological space of the* in silico* target prediction could provide a more comprehensive overview of targets that are involved in the therapeutic effects. Secondly, the chemical space in the chemogenomic database is limited to the version used when the model was developed and in this ChEMBL [49] v.14.0 database, natural compounds only represent approximately 3.85% of the total compounds available [130]. Thus, the limited coverage of chemical space from natural compounds led to zero prediction for targets in the subclass “tranquilizing, settling.” Thirdly, as TCM's therapeutic principle works through* Jun-Chen-Zuo-Shi*, our* in silico* target prediction could only predict the putative targets and the top three enriched targets/pathways might not be modulated by the Emperor compounds of the herb, which play the leading role in treating the disease. In this study, similar top three enriched targets/pathways frequently appeared, which did not represent the actual therapeutic actions described in TCM's philosophy per therapeutic action classes because the definition of the (sub)classes could be limited to the English translation [131]. Although compounds in a TCM formula are known to work synergistically, the algorithm is unable to report whether a compound either activates or inhibits the predicted targets, which can be experimentally influenced by ADMET (Adsorption, Distribution, Metabolism, Excretion, and Toxicity) properties [55]. Therefore, only a general justification could be established between the enriched targets/pathways and therapeutic action (sub)classes.

Despite the limitations, our* in silico* target prediction was able to describe the putative MOA of the compounds from the selected therapeutic action (sub)classes by providing target- and pathway-based MOA hypotheses. With the global overview of the bioactivity space of the therapeutic action (sub)classes, we could observe the similarity and the differences between them, which are not apparent from the name given to the (sub)class itself. Hence, this analysis hopefully helps to bridge the gap between TCM and Western medicine a bit further.

## Supplementary Material

Supplementary Material contains tables of top three enriched targets and pathways in all 14 clusters, in which each enriched target is annotated with its function reported in literature while each enriched pathway is annotated with pathway motif according to KEGG.

## Figures and Tables

**Table 1:** The list of therapeutic action classes, subclasses, and their number of respective Chinese medicines and compounds. A total of 10,749 compounds from 46 therapeutic action subclasses were included in the analysis presented in this work.

Chinese medicine class	Chinese medicine subclass	Chinese medicine (sub)class (Chinese names)	Abb.	NoH	NoC
Exterior releasing	Wind cold dispersing	Sàn hán jiě biǎo yào (*散寒解表药*)	ER-wind cold	21	538
	Wind heat dispersing	Qīng rè jiě biǎo yào (*清热解表药*)	ER-wind heat	22	413

Heat-clearing medicinal	Heat-clearing and blood cooling	Liáng xuè huó xuè yào (*凉血*活*血药*)	HC-blood cool	14	99
	Heat-clearing and dampness drying	Qīng rè lì shī yào (*清热*利*湿药*)	HC-damp	11	264
	Deficiency	Qīng xū rè yào (*清虚热药*)	HC-def	10	186
	Heat-clearing and detoxicating	Qīng rè jiě dú yào (*清热解毒药*)	HC-detox	54	1029
	Heat-clearing and fire purging	Qīng rè xiè huǒ yào (*清热泻*火药)	HC-fire purge	18	234

Purgative medicinal	Laxative medicinal	Rùn xià yào (润下药)	Purg-lax	3	27
	Offensive purgative	Gōng xià yào (攻下药)	Purg-off	6	54
	Drastic (purgative) water-expelling	Jùn xià zhú shuǐ yào (峻下逐水药)	Purg-water expel	14	206

Dampness resolving	Water draining and anti-icteric	Lì shī tuì huáng yào (利*湿退黄药*)	Damp-antiicteric	6	189
	Water draining and strangury resolving	Lì niào tōng lín yào (利*尿通淋药*)	Damp-stran	15	133
	Water draining and swelling dispersing	Lì shuǐ xiāo zhǒng yào (利水*消肿药*)	Damp-swell	13	265

Qi regulating		Lǐ qì yào (理气药)	Qi	36	699

Digestant medicinal		Xiāo shí yào (*消食药*)	Digest	8	146

Hemostatic medicinal	Astringent hemostatic	Shōu liǎn zhǐ xiě yào (*收敛止血药*)	Hemo-astringent	5	92
	Blood cooling hemostatic	Liáng xuè zhǐ xiě yào (*凉血止血药*)	Hemo-blood cool	13	198
	Meridian warming hemostatic	Wēn jīng zhǐ xuè yào (温经*止血药*)	Hemo-meridian	2	146
	Stasis resolving hemostatic	Huà yū zhǐ xiě yào (化*瘀止血药*)	Hemo-stasis	6	245

Blood activating and stasis resolving	Blood activating analgesic	Huó xuè zhǐ tòng yào (活*血止*痛药)	BASR-analgesic	7	487
	Blood breaking mass eliminating	Pò xiě xiāo zhēng yào (*破血消*癥药)	BASR-break	9	177
	Blood activating menstruation resolving	Huó xuè tiáo jīng yào (活*血调*经药)	BASR-menstrual	15	457
	Blood activating trauma curing	Huó xuè liáo shāng yào (活*血疗伤药*)	BASR-trauma	12	261

Cough suppressing and panting-calming	Clearing and heat phlegm resolving	Qīng huà rè tán yào (清化*热痰药*)	CSPC-heat	30	237
	Cold phlegm resolving and warming	Wēn huà hán tán yào (温化*寒痰药*)	CSPC-cold	19	147
	Cough suppressing and panting-calming	Zhǐ ké píng chuǎn yào (*止咳*平*喘药*)	CSPC-panting	16	334

Tranquilizing	Heat nourishing tranquilizing	Yǎng xīn ān shén yào (养心*安神药*)	Tranquil-heat	1	145
	Settling tranquilizing	Zhòng zhèn ān shén yào (*重镇安神药*)	Tranquil-settle	6	1

Orifice opening		Kāi qiào yào (开*窍药*)	Orifice	7	68

Liver-pacifying and wind extinguishing	Extinguishing wind to resolve convulsion	Xí fēng zhǐ jìng yào (*息风止痉药*)	LPWE-convulsion	8	85
	Liver yang calming	Píng yì gān yáng yào (平*抑肝*阳药)	LPWE-liver	7	22

Tonifying and replenishing	Blood tonifying	Bǔ xiě yào (*补血药*)	TR-blood	7	388
	Qi tonifying	Bǔ qì yào (补气药)	TR-qi	15	474
	Yang tonifying	Bǔ yáng yào (补阳药)	TR-yang	23	559
	Yin tonifying	Bǔ yīn yào (补阴药)	TR-yin	17	259

Astringent	Anhidrotic	Gù biǎo zhǐ hàn yào (*固表止汗药*)	Ast-anhidro	3	17
	Lung-intestine astringent	Liǎn fèi sè cháng yào (*敛肺涩肠药*)	Ast-lung	8	145
	Securing essence, reducing urination, and checking vaginal discharge	Gù jīng suō niào zhǐ dài yào (*固精缩尿止带药*)	Ast-secure	6	125

Wind-dampness dispelling	Bone (sinew) strengthening	Qū fēng shī qiáng jīn gǔ yào (*祛风湿强筋骨药*)	WD-bone	5	44
	Heat-clearing	Qū fēng shī rè yào (*祛风湿热药*)	WD-heat	8	175
	Cold dispersing	Qū fēng hán shī yào (*祛风寒湿药*)	WD-cold	13	309

Interior warming		Wēn lǐ yào (温里药)	Warm	13	457

Worm expelling medicinal		Qū chóng yào (驱虫药)	Worm	9	93

Emetic medicinal		Yǒng tǔ yào (*涌吐药*)	Emetic	3	9

Parasite destroying, dampness eliminating, and itchiness relieving		Gōng dú shā chóng zhǐ yǎng yào (*攻毒杀*虫*止痒药*)	Parasite	8	81

Antimalarial medicinal		Kàng nüè yào (*抗疟药*)	Malarial	4	30

			Total compounds		10,749

Abb.: abbreviation.

NoH: number of Chinese medicines.

NoC: number of compounds.

**Table 2:** The top three enriched targets in clusters VII, X, and XII. It can be seen that, in many cases, the top three enriched targets are implicated in immunomodulation. Estimation Score = 0, for all top three enriched targets.

	TCM therapeutic action class	TCM therapeutic action subclass	Top three enriched targets	Target function reported by literatures	Average Score
Cluster X	Wind-dampness dispelling	Bone (sinew) strengthening	DNA topoisomerase 1	Cancer	0.0144
			Sodium/glucose cotransporter 1	Glucose homeostasis	0.0342
			Steryl-sulfatase	Immunomodulation	0.0370
	Tonifying and replenishing	Qi tonifying	Tyrosine-protein phosphatase nonreceptor type 2	Immunomodulation	0.0174
			Sodium/glucose cotransporter 2	Glucose homeostasis	0.0282
			Sodium/glucose cotransporter 1	Glucose homeostasis	0.0321
	Cough suppressing and panting-calming	Clearing and heat phlegm resolving	Tyrosine-protein phosphatase nonreceptor type 2	Immunomodulation	0.0112
			DNA topoisomerase 1	Cancer	0.0236
			Testosterone 17-beta-dehydrogenase 3	Reproduction system	0.0341
	Tranquilizing	Heat nourishing tranquilizing	Peptidyl-prolyl cis-trans isomerase FKBP1A	Immunomodulation	0.0077
			Tyrosine-protein phosphatase nonreceptor type 2	Immunomodulation	0.0167
			Glutamate carboxypeptidase 2	CNS	0.0209

Cluster VII	Heat-clearing medicinal	Heat-clearing and blood cooling	Protein kinase C beta type	Immunomodulation	0.0100
			DNA topoisomerase 1	Cancer	0.0123
			Sodium/glucose cotransporter 2	Glucose homeostasis	0.0137
	Hemostatic medicinal	Stasis resolving hemostatic	Tyrosine-protein phosphatase nonreceptor type 2	Immunomodulation	0.0089
			Protein kinase C eta type	Immunomodulation	0.0182
			Protein kinase C gamma type	Immunomodulation	0.0209
	Tonifying and replenishing	Blood tonifying	Tyrosine-protein phosphatase nonreceptor type 2	Immunomodulation	0.0140
			Protein kinase C beta type	Immunomodulation	0.0230
			Protein kinase C eta type	Immunomodulation	0.0240

Cluster XII	Parasite destroying, dampness eliminating, and itchiness relieving		Dihydrofolate reductase	Cancer, bacterial infection	0.0532
			DNA-dependent protein kinase catalytic subunit	Cancer	0.0644
			Tumour necrosis factor	Cancer, bacterial infection	0.0687

**Table:** (a) Cluster X

Therapeutic action (sub)class	Herb	Compound	Predicted targets (based on the top three enriched targets)	Literature support
Wind-dampness dispelling, bone strengthening (WD-bone)	*Acanthopanax gracilistylus*	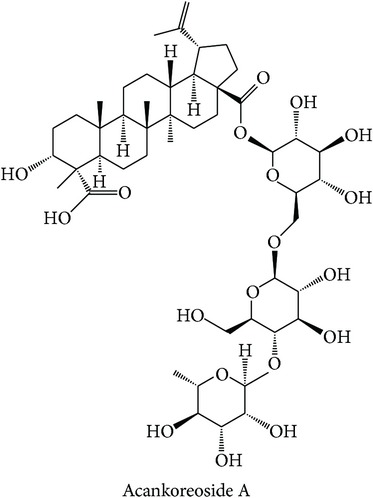	TOPO1 SGLT1	The herb's extract inhibited cell proliferation of several types of cancer cells [72].

Tonifying and replenishing, qi tonifying (TR-qi)	*Glycyrrhiza glabra* *Glycyrrhiza uralensis*	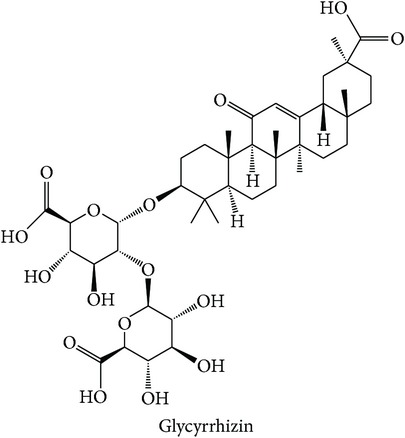	PTPN2	An *in vivo* study suggested the compound ameliorates all established chronic histopathologic changes of lung in the mouse model of asthma [78].

Cough suppressing and panting-calming, clearing and heat phlegm resolving (CSPC-heat)	*Platycodon grandiflorum*	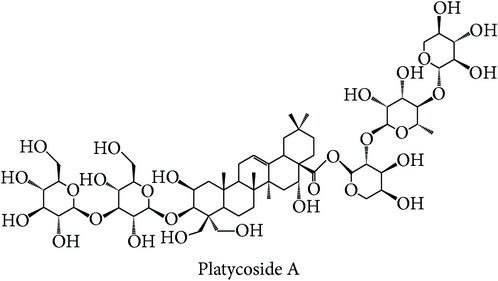	PTPN2 TOPO1 17-beta-HSD 3	There is no supporting literature.

Tranquilizing, heart nourishing tranquilizing (Tranquil-heart)	*Ganoderma lucidum*	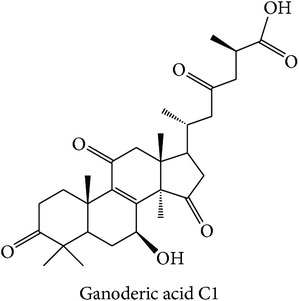	CGPII	Triterpenoids of the herb exhibited nerve growth factor or brain-derived neurotrophic factor activities *in vitro*, which has the therapeutic potential in neurodegenerative diseases [81].

**Table:** (b) Cluster VII

Therapeutic action (sub)class	Herb	Compound	Predicted targets (based on the top three enriched targets)	Literature support
Hemostatic, stasis resolving (Hemo-stasis)	*Rubia cordifolia*	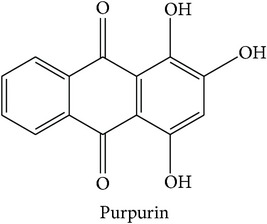	PKC-*β* PKC-*ε*	The ethanol extract showed wound healing activities in mice, which from histological evaluations indicated marked infiltration of the inflammatory cells, increased blood vessel formation, and enhanced proliferation of cells [85].

Tonifying and replenishing, blood (TR-blood)	*Panax notoginseng*	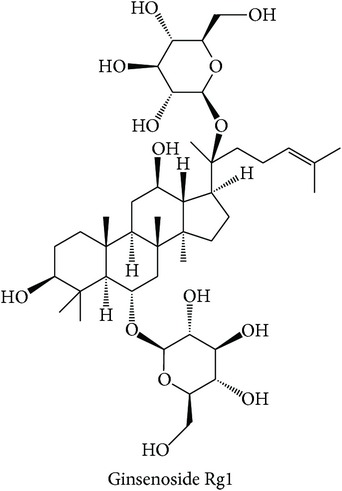	PTPN2 PKC-*η*	The compound ameliorated liver damage and suppressed proinflammatory cytokines secretion in concanavalin A-induced hepatitis in mice [86].

Heat-clearing, blood cooling (HC-blood cool)	*Rehmannia glutinosa*	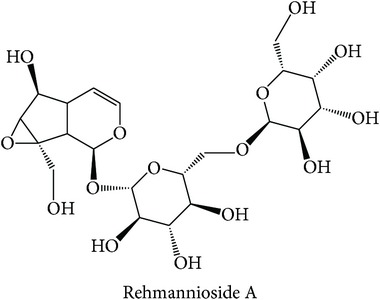	SGLT2	The stachyose extract from the herb showed a significant hypoglycaemic effect in diabetic mice [87].

**Table:** (c) Cluster XII

Therapeutic action (sub)class	Herb	Compound	Predicted targets (based on the top three enriched targets)	Literature support
Parasite destroying, dampness eliminating, and itchiness relieving (Parasite)	*Allium sativum*	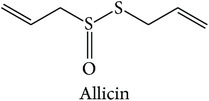	DHFR	The extract of *Allium sativum* was found to inhibit the synthesis of proteins, nucleic acids, and lipids in *Candida albicans* where the major component of the herb was allicin [94].

**Table 4:** Compound from CHEMBL database that is most similar to acankoreoside A (Table 3) and its activity profile.

Reference compound	Closest similarity	Reported activity profile
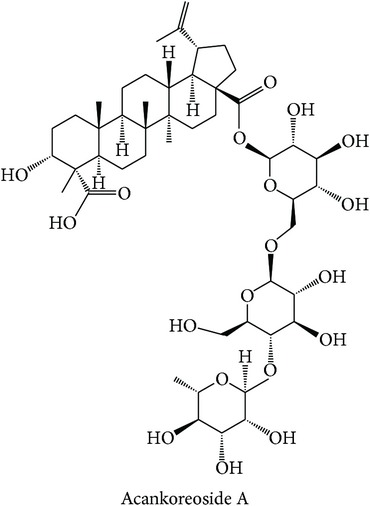	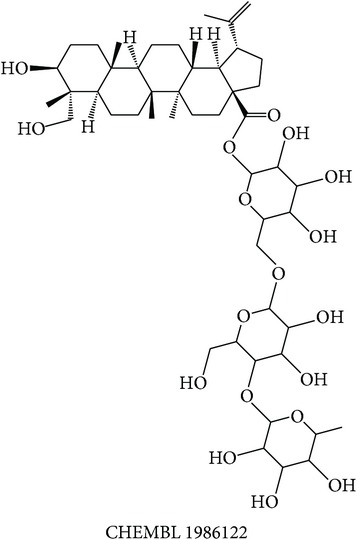	The GI_50_ value of CHEMBL 1986122 was 43.05 nM in CCRF-CEF leukaemia cell line growth inhibition assay. *Tanimoto coefficient* = 0.91

**Table 5:** The top three enriched pathways in clusters VII, X, and XII. It can be seen that, in many cases, similar pathways appear in the top three enriched targets regardless of clusters and subclasses. Estimation Score = 0, for all top three enriched pathways.

	TCM therapeutic action class	TCM therapeutic action subclass	Top three enriched pathways	Average Score
Cluster X	Wind-dampness dispelling	Bone (sinew) strengthening	hsa04978, mineral absorption	0.0342
			hsa04973, carbohydrate digestion and absorption	0.1427
			hsa04976, bile secretion	0.2050
	Tonifying and replenishing	Qi tonifying	hsa04978, mineral absorption	0.0321
			hsa04973, carbohydrate digestion and absorption	0.1251
			hsa04976, bile secretion	0.2678
	Cough suppressing and panting-calming	Clearing and heat phlegm resolving	hsa04978, mineral absorption	0.0380
			hsa04973, carbohydrate digestion and absorption	0.1572
			hsa04976, bile secretion	0.1958
	Tranquilizing	Heat nourishing tranquilizing	hsa04978, mineral absorption	0.0387
			hsa04973, carbohydrate digestion and absorption	0.1163
			hsa00900, terpenoid backbone biosynthesis	0.2166

Cluster VII	Heat-clearing medicinal	Heat-clearing and blood cooling	hsa04978, mineral absorption	0.0276
			hsa04973, carbohydrate digestion and absorption	0.0753
			hsa02010, ABC transporters	0.0769
	Hemostatic medicinal	Stasis resolving hemostatic	hsa04978, mineral absorption	0.0267
			hsa04973, carbohydrate digestion and absorption	0.0987
			hsa04976, bile secretion	0.1872
	Tonifying and replenishing	Blood tonifying	hsa04978, mineral absorption	0.0329
			hsa04973, carbohydrate digestion and absorption	0.1055
			hsa04530, tight junction	0.2053

Cluster XII	Parasite destroying, dampness eliminating, and itchiness relieving		hsa00100, steroid biosynthesis	0.253
			hsa00564, glycerophospholipid metabolism	0.294
			hsa04966, collecting duct acid secretion	0.342
